# MP-LAMP: parallel detection of statistically significant multi-loci markers on cloud platforms

**DOI:** 10.1093/bioinformatics/bty219

**Published:** 2018-04-06

**Authors:** Kazuki Yoshizoe, Aika Terada, Koji Tsuda

**Affiliations:** 1 Center for Advanced Intelligence Project, RIKEN, Tokyo, Japan; 2 Department of Computational Biology and Medical Sciences, Graduate School of Frontier Sciences, The University of Tokyo, Kashiwa, Japan; 3 PRESTO, Japan Science and Technology Agency, Kawaguchi, Japan

## Abstract

**Summary:**

Exhaustive detection of multi-loci markers from genome-wide association study datasets is a computationally challenging problem. This paper presents a massively parallel algorithm for finding all significant combinations of alleles and introduces a software tool termed MP-LAMP that can be easily deployed in a cloud platform, such as Amazon Web Service, as well as in an in-house computer cluster. Multi-loci marker detection is an unbalanced tree search problem that cannot be parallelized by simple tree-splitting using generic parallel programming frameworks, such as Map-Reduce. We employ work stealing and periodic reduce-broadcast to decrease the running time almost linearly to the number of cores.

**Availability and implementation:**

MP-LAMP is available at https://github.com/tsudalab/mp-lamp.

**Supplementary information:**

Supplementary data are available at *Bioinformatics* online.

## 1 Introduction

In the last decade, a large number of genome-wide association studies (GWASs) have been conducted and created a substantial resource of genomic data. It is often pointed out that this data resource has not been fully exploited because most genetic studies investigate the effect of a single-locus marker only (Niel *et al.*, 2015). Finding multi-loci markers involving three loci or more presents a tremendous computational challenge due to combinatorial explosion. It also poses a statistical problem that the probability of occurrence of at least one false discovery, called the family-wise error rate (FWER), increases as the number of marker candidates increases.

A multiple testing procedure, such as Bonferroni correction, is used to solve this serious statistical problem, but most procedures calculate the upper bound of FWER too loosely and the calculated correction factor is very large in multi-loci marker analysis. To reduce the correction factor by a tighter bound of FWER, Terada *et al.* proposed a tree-search algorithm, called Limitless Arity Multiple-testing Procedure (LAMP; Terada *et al.*, 2013), by using the following property (Tarone, 1990). Given a set of multi-loci markers to be evaluated for association with the phenotype, the multi-loci markers can be divided into *testable* and *untestable* ones. Testable markers have the possibility of causing a false positive result, while untestable ones have no possibility of doing so. Therefore, we only count the number of testable ones in the correction factor.

Despite these theoretical advantages, the application of LAMP to GWAS is still confined to a few studies. It is known that LAMP requires substantially more computational time if SNPs of high minor allele frequency (MAF) are included in the data (Terada *et al.*, 2016).

Recently, cloud platforms such as Amazon Web Services (AWS) have emerged as a cost-effective alternative to in-house computer clusters. By default, AWS offers Map-Reduce as a parallel processing framework. However, LAMP cannot be parallelized simply by Map-Reduce because the algorithm is designed using an unbalanced tree search. We therefore implemented a software called MP-LAMP, which is directly built on Message Passing Interface (MPI). Applied to the GWAS datasets, the running time of MP-LAMP decreased almost linearly relative to the number of cores.

## 2 Implementation and usage

We parallelized a depth-first search algorithm (Minato *et al.*, 2014) to overcome time-consuming problem because it is the fastest algorithm of LAMP on a single core. Our code is implemented in C and uses three libraries: MPI Library, boost library ≥ 1.55.0 and gflags ≥ 2.0. We here describe the key ideas of MP-LAMP. The detailed algorithms are described in the Supplementary Material.

Let us call a computing entity *worker*. Parallelization of LAMP has to satisfy the following three requirements. (i) Workers should traverse the search tree collectively without load unbalance. (ii) The threshold $\lambda^{\prime }$ for the frequency of multi-loci markers is shared by all workers because it gradually increases during the depth-first search. (iii) Exact number of frequent multi-loci markers with frequency higher than $\lambda^{\prime }$ is counted.

**Fig. 1.**
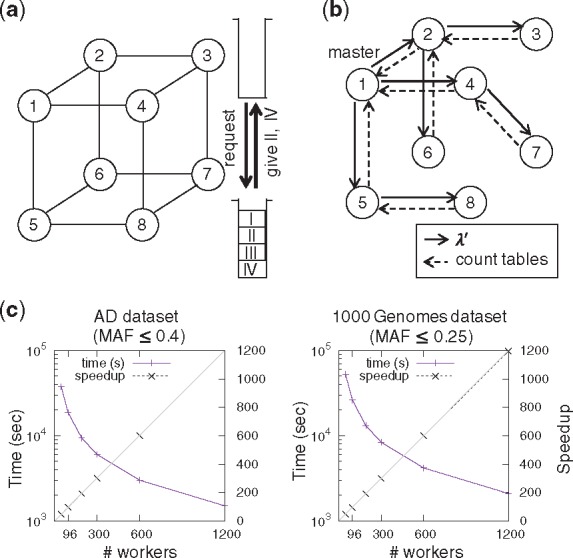
Strategies of MP-LAMP and time performance. (**a**) Work stealing. A hypercube communication graph is used for low-overhead task distribution. A vertex and edge represent a worker and communication between them, respectively. (**b**) Reduce-broadcast. The communication graph is a rooted spanning tree. (**c**) Running time and speedup with increasing number of workers

To fulfill the first requirement, *work stealing* on hypercube topology (Saraswat *et al.*, 2011) is employed for the co-ordinated traversal. It is conducted with communication between the adjusted workers in a de-centralized manner. Figure 1a shows an example. Worker 3 has no jobs in the stack, while worker 7 has four jobs I to IV. Therefore, worker 3 sends a request message to worker 7 to get jobs and worker 7 gives half of the jobs as a reply.

For the second and third requirements, we develop *reduce-broadcast* (Mattern, 1990). Unlike the work stealing, the reduce-broadcast defines the master worker in advance as shown in Figure 1b. First, the master worker sends $\lambda^{\prime }$ to its child nodes (the solid lines). When a leaf worker receives $\lambda^{\prime }$, it sends back a reply message to its parent (the dotted lines). These reply messages include the count table, which keeps the number of multi-loci markers that are found. Finally, the master worker obtains the total count table that allows it to update the threshold $\lambda^{\prime }$.

MP-LAMP is invoked with the following command: $mpiexec -np [# workers]./mp-lamp –item [file1] –pos [file2]. The number of workers is set with the -np option of the mpiexec command. The two csv files are given with –item and –pos. The former file represents single-marker genotypes of each sample. The latter file gives the phenotype of each sample. Additionally, the significance level can be changed by using thea–a option.

The outputs include statistically significant multi-loci markers whose *P*-value is smaller than the adjusted significance level. By default, one-sided Fisher’s exact test is selected to calculate the *P*-value. This setting can be changed by using the -*P* and –alternative options. Additional descriptions of these options are shown in Supplementary Table S1.

## 3 Results

We applied MP-LAMP to two GWAS datasets. One dataset is human exome data provided by the 1000 Genomes Project (The International HapMap Consortium, 2005; 1000 genomes dataset). The other dataset was obtained from an Alzheimer GWAS study (Webster et al., 2009; AD dataset). We used cSNPs in these data. The former dataset consists of 12 758 SNPs with 105 cases and 592 controls. The latter dataset has 3307 SNPs with 176 cases and 188 controls. One-sided Fisher’s exact test was used to calculate the *P*-value. The significance level was set to 0.05.

Experiments were performed on two environments: cloud computing with the AWS and a High-Performance Computing cluster. The detailed environments are shown in Supplementary Table S2.

We evaluated the performance of MP-LAMP using GWAS datasets that contains SNPs with low MAF because the original LAMP can be slow if SNPs with high MAF are included. The MAF threshold and the number of SNPs analyzed are shown in Supplementary Tables S3 and S4. Supplementary Figure S1 shows that parallelization algorithm works efficiently on cloud computing of AWS. The running time of MP-LAMP linearly decreases with an increasing number of workers when the dataset contains many SNPs (Supplementary Fig. S1c and g).

When MP-LAMP was run on a massively parallel cluster, it achieved almost linear speedup as the number of cores increased. Analysis of the AD dataset containing SNPs with MAF ≤0.4 finished in 1504.585 s with 1200 workers (Fig. 1c). Without parallel computation, it was estimated that over 20 days would be required to conduct an identical analysis. The results of the 1000 genomes dataset were similar to these for the AD dataset analysis (Supplementary Fig. S2). These results indicate that our parallelization algorithm works efficiently to reduce the running time in a GWAS analysis even when over 1000 workers are used. We also evaluated the influence of MAF threshold in computational time. The result is summarized in Supplementary Figure S6.

Our analysis of the AD dataset yielded numerous statistically significant markers including three or more SNPs. When the AD dataset was analyzed with a MAF ≤ 0.4, 651 markers were detected as statistically significantly associated with case individuals. Among them, 552 markers consisted of at least three SNPs, and the largest markers include five SNPs. All of the significant markers are listed in Supplementary Table S7. Our results include multi-loci markers that have been confirmed to be associated with AD development, as described in Supplementary Material.

## Funding

This work was supported by KAKENHI 25700038, 15H02708 to K.Y. JST PRESTO to A.T. JST CREST JPMJCR1502, RIKEN PostK and KAKENHI 15H05711 to K.T.

*Conflict of Interest*: none declared.

## Supplementary Material

Supplementary Data
