# Supplementary material for: MP-LAMP: parallel detection of statistically significant multi-loci markers on cloud platforms
Source: Bioinformatics. 2018 Apr 6;34(17):3047–9. doi: 10.1093/bioinformatics/bty219 (PMC6129301; doi:10.1093/bioinformatics/bty219)
Supplement: Supplementary Data [file bty219_supp.zip › bty219-suppl_data/mplamp_supp.pdf]

Supplementary Text for "MP-LAMP: Parallel Detection of Multi-Loci Markers on Cloud Platforms"

Kazuki Yoshizoe[1,2], Aika Terada[3,2], and Koji Tsuda[2,1]

[1]Advanced Intelligence Project, RIKEN
[2]Department of Computational Biology and Medical Science, The University of Tokyo
[3]PRESTO, Japan Science and Technology Agency

S1 Supplementary Tables

Table S1: Options for MP-LAMP. The bold letters indicate the required options.

Option	Description
**--item**	Input filename giving genotypes of samples
**--pos**	Input filename giving phenotype of samples
--a	Set a statistical significance level (default is 0.05)
-p	Selecting which statistical method is used from "fisher" (Fisher's exact test), "chi" (chi-squared test), or "u_test" (Mann-Whitney U test). Default is fisher.
--alternative	Selecting which alternative hypothesis is used from "greater", "less", or "two.sided". The default setting is "greater".

Table S2: Two environments used to evaluate time performance.

(a) AWS (c4.8xlarge instance)

OS	CPU	# cores	Memory	interconnect
Amazon Linux AMI 2016.03.3 (HVM)	Intel Xeon E5-2666v3 (18 cores, 2.9 GHz)	18	60 GiB	10G Ethernet

(b) HPC cluster 1 (TSUBAME at Tokyo Institute of Technology)

OS	CPU	# cores	Memory	interconnect
SUSE Linux Ent. Server 11 SP3	Intel Xeon X5670 (6 cores, 2.9 GHz)	12	52 GiB	InfiniBand QDR dual-rail (40Gbps)

(c) HPC Cluster 2 (RAIDEN PC cluster subsystem at RIKEN AIP)

OS	CPU	# cores	Memory	interconnect
Cent OS 7.2	Intel Xeon E5-2690v4 (14 cores, 2.6 GHz)	28	256 GiB	InfiniBand EDR 100Gbps

Table S3: Results of 1000 genomes dataset analysis. This dataset contains 105 cases and 592 controls.

MAF upper limit	# SNPs (%)	Correction factor	Support	# markers detected	Max. size	Time (1 core)	Time (72 cores)
0.10	11,253 (88.2 %)	90,999	8	92	5	59.612s	2.410s
0.15	11,676 (91.5 %)	3,004,701	10	306	5	30m 0s	31.003s
0.20	11,914 (93.4 %)	47,835,176	11	1,178	8	7h 2m 51s	6m 39s
0.25	12,124 (95.0 %)	2,196,662,623	13	1,475	9	15d 9h 32m 48s†	5h 48m 32s

† Running time was estimated from the result using eight cores.

Table S4: Results of AD dataset analysis. This dataset contains 176 cases and 188 controls.

MAF upper limit	# SNPs (%)	Correction factor	Support	# markers detected	Max. size	Time (1 core)	Time (72 cores)
0.10	902 (27.3 %)	2,403	15	0	-	0.183s	0.0980s
0.20	1,681 (50.8 %)	301,067	21	0	-	26.557s	0.799s
0.30	2,287 (69.1 %)	35,156,598	27	290	5	1h 1m 38s	1m 6s
0.40	2,808 (84.9 %)	7,441,684,271	34	651	5	11d 12h 50m 16s†	4h 11m 20s

† Running time was estimated from the result using eight cores.

S2 Supplementary Figures

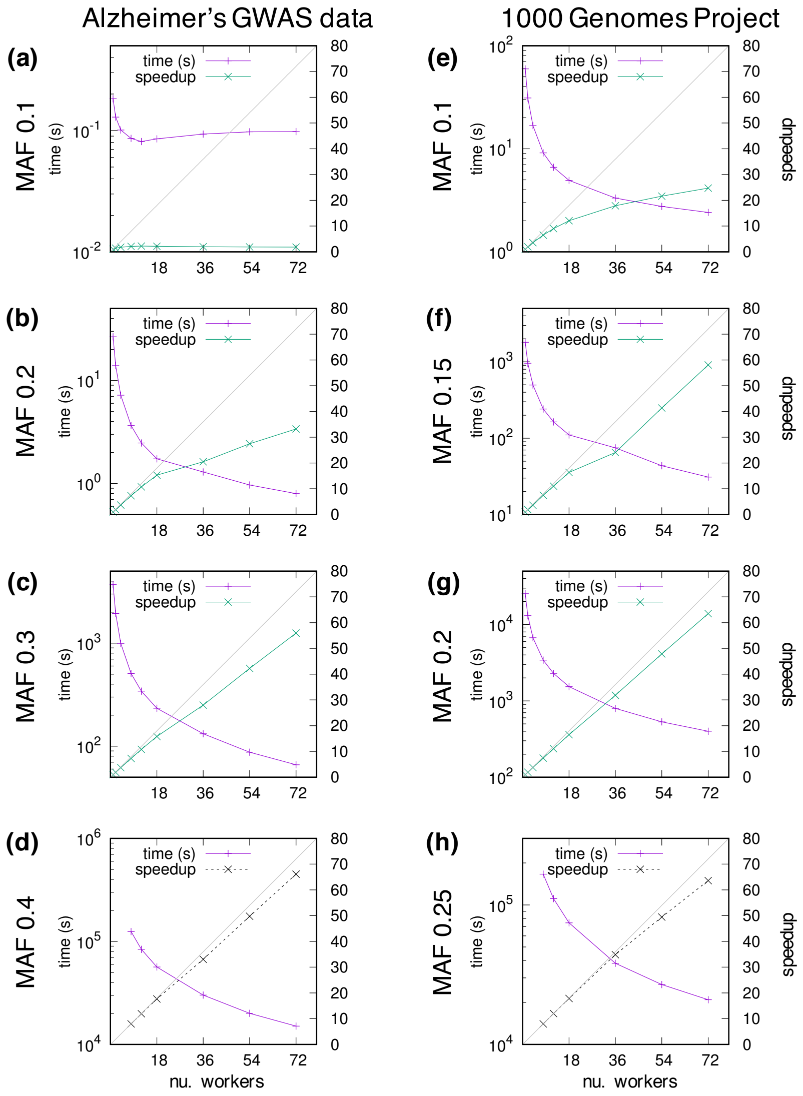

Figure S1: Running time and speedup with an increasing number of workers on a cloud cluster.

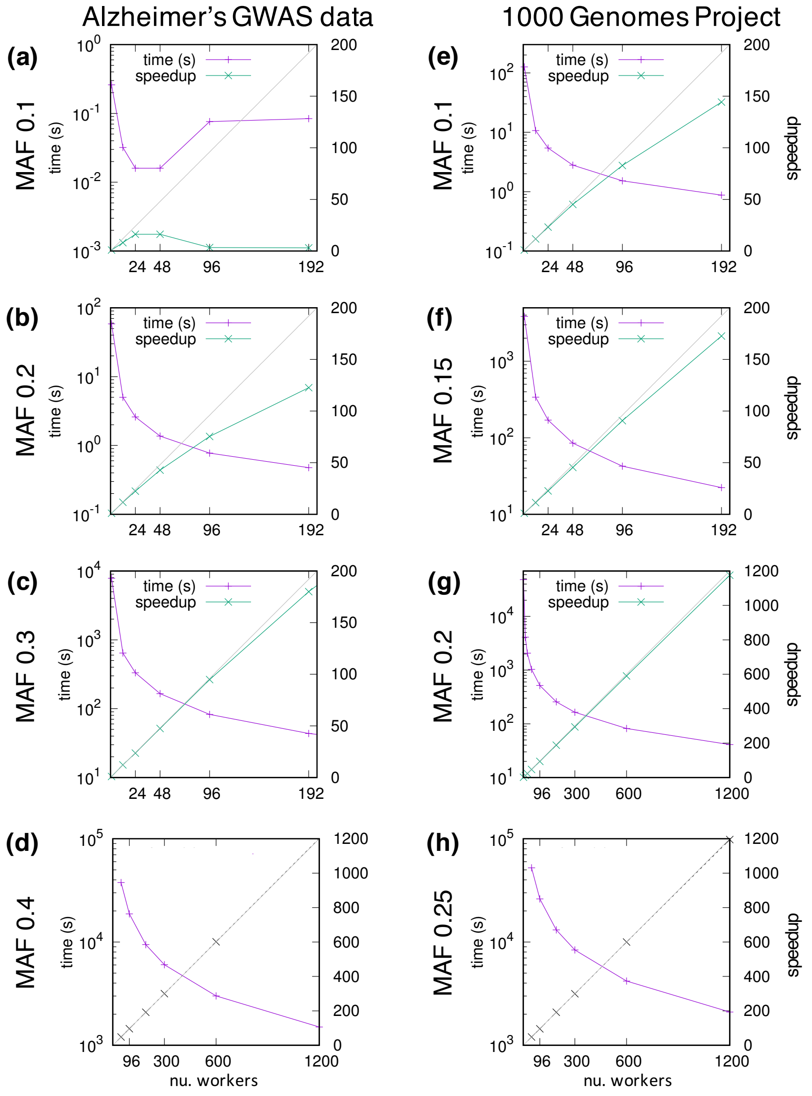

Figure S2: Running time and speedup with an increasing number of workers on an HPC cluster.

S3 LAMP

Most existing tools for GWAS only focus on singles or pairs of SNPs. Ideally, it should be extended to combinations of n SNPs, which we call *multi-loci markers*. Given a dataset in Table S5, the contingency table of a multi-loci marker g_1, g_4, g_6 is shown in Table S6, from which a p-value is derived using, e.g., Fisher exact test. By computing p-values for all $2^n - 1$ combinations and correcting them by a multiple testing procedure, we can find all significant multi-loci markers, but it is possible only for very small datasets. LAMP [10] is an efficient algorithm to detect all significant multi-loci markers without visiting all combinations. It is possible because of the following theoretical reasons. 1) Multiple markers result in an exactly same contingency table. 2) The support (i.e., frequency) of most markers is very small.

To exploit the first point, all possible contingency tables are listed up with closed itemset mining [13]. A closed marker for a contingency table is defined as the maximal one among those resulting in the table. The tree consisting of closed markers only is shown in Figure S3. LAMP is based on an efficient algorithm called LCM [13] that discovers all closed markers by using *prefix preserving closure extension*, i.e., first add an item to a current set and then take the closure operation.

The second point can be exploited by a family of discrete tests such as Tarone test [9] that reduce the correction factor by ignoring hypotheses with small support. For example, Tarone test exploits the fact that the p-value of a multi-loci marker with support x cannot be smaller than

$$f(x) = \begin{cases} \binom{N_1}{x} \Big/ \binom{N}{x} & (x \leq N_1) \\ 1 \Big/ \binom{N}{x} & (otherwise), \end{cases} \tag{S1}$$

where N and N_1 represent the numbers of all samples and those with the positive phenotype, respectively. During the tree search of LAMP, a lower bound of the number of significant multi-loci markers is constantly updated. By combining the lower bound and the p-value bound (S1), LAMP can identify unnecessary parts of the tree and prune them out.

There are two algorithms for LAMP. The original algorithm [10] employs step-wise search and is not efficient. The new efficient algorithm by Minato et al. [6] employ a stack and perform depth-first search. During the search, LAMP keeps track of a table recording the current number of markers whose support is at least λ, $\kappa'(\lambda)$ (Figure S4). Using the table, the pruning threshold λ' is calculated and backtracking happens when a marker whose support is smaller than λ' is found.

S4 MP-LAMP

We parallelize LAMP by distributing the stack used in depth-first search to multiple workers. A worker can be a CPU core, a process or a thread. Each worker i has its

Table S5: Example dataset. This dataset contains seven samples $\{s_1, \cdots, s_7\}$ and genotypes of six SNPs $\{g_1, \cdots, g_6\}$

Sample	Genotype						Phenotype
	g_1	g_2	g_3	g_4	g_5	g_6	o
s_1	1	1	1	1	1	1	0
s_2	0	1	1	0	1	0	0
s_3	0	1	0	0	1	0	1
s_4	1	1	0	1	1	1	1
s_5	1	1	0	1	0	0	0
s_6	1	0	0	1	0	1	1
s_7	0	0	1	1	0	1	0

Table S6: The contingency table to calculate the p-value of marker $G = \{g_1, g_4, g_6\}$.

	$g_1 = 1$, $g_4 = 1$ and $g_6 = 1$	Others	Total
$o = 1$	2	1	3
$o = 0$	1	3	4
Total	3	4	7

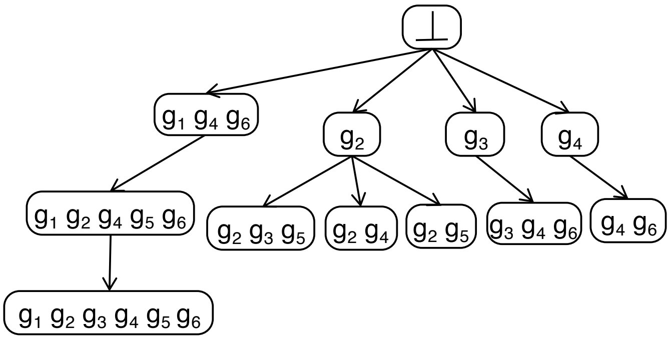

Figure S3: The search tree of all closed markers derived from the dataset in Table S5.

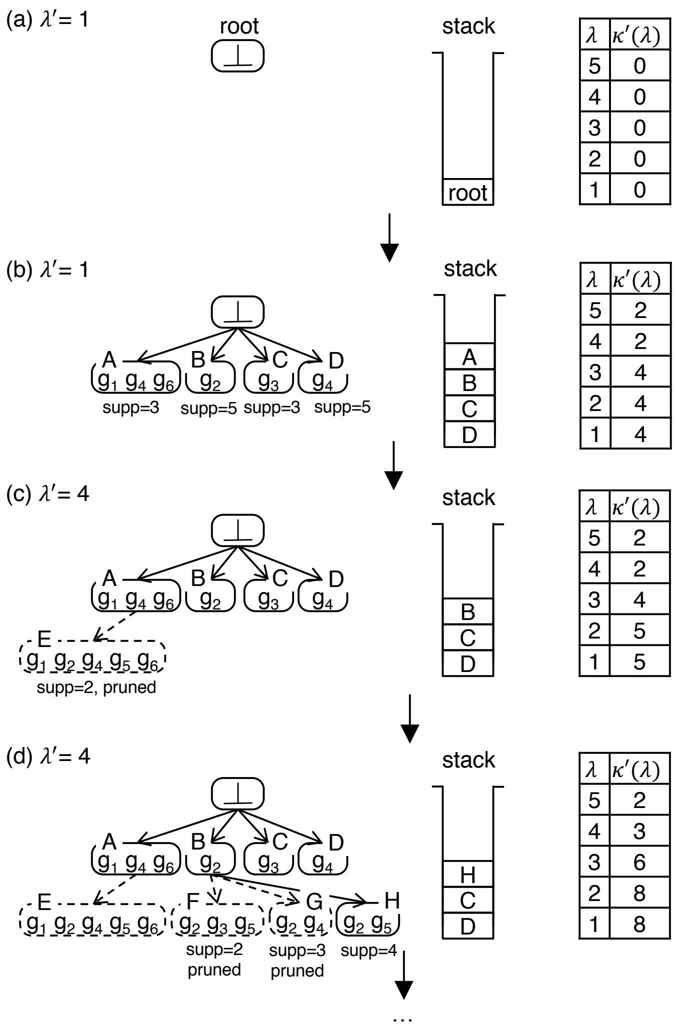

Figure S4: Depth-first search using a stack. As search progresses, the count table for keeping the number of markers of different support is updated.

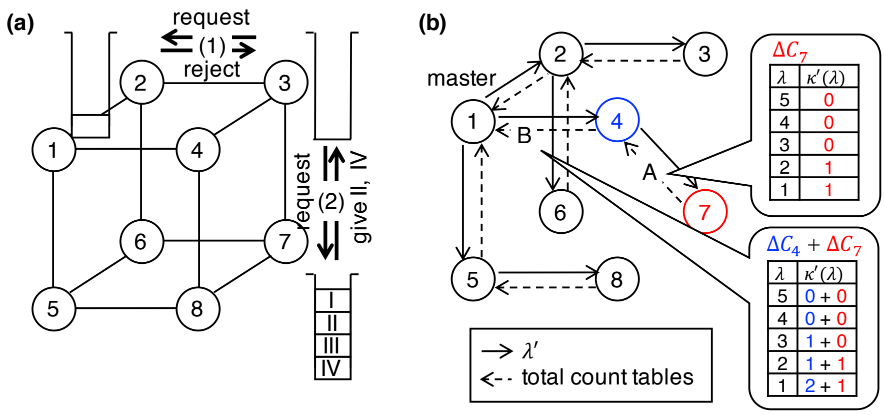

Figure S5: Two methods used in MP-LAMP. (a) Work stealing. A hypercube communication graph is used to evenly distribute tasks to the workers. A vertex and edge represent a worker and communication between them, respectively. Worker 3 sends the requests to adjacent workers to get tasks. (b) Reduce-broadcast. The workers communicate with each other along a rooted spanning tree. The master worker broadcasts λ' to child nodes (solid arrows). Upon arrival of the replies from the children (dotted arrows), each worker sums up the received count tables and its own one, and then sends the total count table to the parent.

own stack S_i to keep assigned nodes of the search tree. In addition, each worker i counts closed markers and keeps them into their own count table C_i. Note that C_i is in non-shared memory and other workers cannot directly refer to the content.

The following three requirements must be fulfilled for efficient parallelization. 1) Workers traverse the search tree collectively without workload imbalance. 2) The threshold λ' is shared by all workers. 3) Count tables of all workers must be summarized for updating λ'. We employ the *work stealing* method [7] to solve the first requirement. For the other requirements, we used a modified version of the *reduce-broadcast* method [1].

S4.1 Work Stealing

In initialization, children of the root node (i.e., depth 1 nodes) are distributed to the stacks of all workers. Then, each worker i performs the tree search by repeatedly popping a node from S_i, creating the child nodes, and pushing them to S_i. When S_i becomes empty, the worker i sends request messages to another worker (the victim) for stealing nodes from the stack. If the target victim has more than two nodes, it sends half of its nodes to the stealer. Otherwise, the request is rejected.

Figure S5 (a) shows this procedure. In this figure, worker 3 tries to steal nodes from adjacent workers because its stack is empty. First, worker 3 sends a request to worker 2 (communication 1). Worker 2 does not have enough nodes to share, and hence it sends back a reject message to worker 3. After getting this reply from worker 2, worker 3 sends a request to another worker 7 (communication 2). Worker 7 gives half of its nodes as a reply. These processes are conducted until the stacks of all workers become empty.

If we allow a worker to send request messages to all of the other workers as frequently as possible, the workers would be interrupted too frequently. Therefore, we employ the following strategy proposed by Saraswat et al. [7]. First, a worker sends requests w times to random workers. If all random requests fail, a worker sends requests to adjacent workers on a hypercube communication graph. If they also fail, the worker sleeps after informing the adjacent workers. When one of the adjacent workers turns out to have more than two markers, it gives a half of them to the sleeping worker so that it can restart working.

S4.2 Reduce-broadcast

For the second and third requirements, we utilize the reduce-broadcast [1]. Our strategy consists of two steps. 1) The master worker informs λ' to all workers. 2) Each worker replies by sending the count table. Figure S5 (b) represents the reduce-broadcast procedure. It begins when the (randomly chosen) master worker sends λ' to its child nodes (the solid lines). The communication graph for this procedure is a rooted spanning tree. The child worker who received an exploration message further propagates the message to its children.

When an exploration message arrives at a leaf worker, it sends back an echo message to its parent (edge A). The echo message contains ΔC_i, which represents the increment of count table C_i of the leaf worker i. As a worker node receives the echo messages from

all of its children (on the rooted spanning tree communication graph), it sums up all the count tables, creates a new echo message and sends it to its parent (edge B). Finally, the master worker obtains the total count table that allows it to update the threshold λ'. Information necessary for distributed termination detection [5] is also conveyed in exploration and echo messages. This reduce-broadcast is performed periodically (e.g., at most once every 1ms).

S5 Analysis Setting

We applied our parallel method to two GWAS datasets for evaluating the computational efficiency. One dataset is human exome data provided by the 1000 Genomes Project [12] (1000 genomes dataset). We prepared a case-control dataset by regarding Japanese individuals as cases and the others as controls. See the supplementary report of [11] for the command lines to create the dataset. The other dataset was obtained from an Alzheimer GWAS study [14] (AD dataset). We used cSNPs (SNPs in coding regions of genes where the different SNP alleles code for different amino acids in the protein) in these data. The former dataset consists of 12,758 SNPs, with 105 cases and 592 controls. The latter dataset has 3,307 SNPs, with 176 cases and 188 controls.

We evaluated an association between a marker and the phenotype by *dominant exclusive model* [11]. Given a marker of a biallelic SNP, three genotypes can be considered: dominant homozygote, heterozygote, and recessive homozygote. The dominant exclusive model considers that heterozygote and recessive homozygote increase the risk of disease. Similarly, for a marker of multiple SNPs, when all SNPs of the marker are heterozygote and recessive homozygote, it is considered to promote the disease development. The statistical significance of a marker is evaluated with the one-sided Fisher's exact test, and the significance level is set to 0.05.

MP-LAMP is designed as a general purpose tool. LAMPLINK can be used for converting GWAS data to MP-LAMP input. In this experiment, we used the dominant exclusive model, but one can choose other models such as recessive model. Also, MP-LAMP supports one- and two-sided Fisher's exact test, one- and two-sided chi-squared tests, and Mann-Whitney U test.

Experiments are performed on (i) commodity cloud computing server with the Amazon Web Service (AWS) and (ii) High-Performance Computing (HPC) cluster equipped with a high speed interconnect. The experiments on a cloud platform were run on c4.8xlarge instances of the AWS (specs in Table S2 (a)). For the HPC cluster, we had used TSUBAME supercomputer at Tokyo Institute of Technology (specs in Table S2 (b)). The number of random steal w is set to one. The length of the edges the hypercube communication graph is set to two, and the dimension was defined accordingly (e.g. for 32 workers, dimension is set to 5). For the rooted spanning tree graph, a ternary tree is constructed.

S6 Significant Markers Associated with Alzheimer's Disease

Our analysis of the AD dataset yielded numerous statistically significant markers including three or more SNPs. When the AD dataset was analyzed with an minor allele frequency (MAF) threshold of 0.4, 651 markers were detected as being statistically significantly associated with case individuals. More than 552 markers consisted of three SNPs, and the largest markers included five SNPs. All of the significant markers are listed in Supplementary Table 7.

All significant markers include rs429358, which is a well-known causal factor of Alzheimer's disease (AD). Variation of rs429358 genotype produces Apolipoprotein E (ApoE) isoforms, ApoE2, 3, and 4, and ApoE4 is associated with an increasing risk of AD [2]. We also detected a single marker composed of rs429358 (COMB 542 in Supplementary Table 7), but its OR was not so high compared with those of other significant markers.

The highest OR marker in Supplementary Table 7 (COMB 1) includes SNPs located on *MTOR* or *HSPA1L*, which produce mammalian target of rapamycin (mTOR) or Heat Shock Protein Family A (Hsp70). According to literature, interaction between mTOR and Hsp70 associates with pathological processes of AD. Hsp70 induced inhibition of autophagy by activation of mTOR [8, 3], and defective autophagy is considered to be a primary factor contributing to AD pathogenesis [15, 4]. Although further biological validation is necessary, this result may suggest possible roles of MTOR and HSPA1L in AD.

S7 Experiments with and without MAF filtering

Generally for a search algorithm, it is difficult to predict the runtime without actually trying. To analyze the difficulty, we had compared the runtime for several datasets (Figure S6). The X-axis shows the upper limit of MAF and the Y-axis shows the runtime.

The datasets are prepared based on the AD dataset using the dominant model. The two black lines are for the whole genome data (380,157 SNPs) and the cSNPs (3,309 SNPs). Then we had randomly extracted 1000, 10000, 100000 SNPs from the whole genome dataset to prepare three more datasets. Please note that the number of SNPs (shown beside each plot) decreases as MAF limit decreases.

The runtime was measured using 25 compute nodes ($25 \cdot 28 = 700$ CPU cores) of the RAIDEN cluster described in Table S2 (c). The parameters of the parallel algorithms are the same as in Section S5. As expected, we can see that the runtime drastically increases if the size of the datasets increases. It can also be seen that the runtime increases for higher MAF limit by comparing the plots along horizontal lines. It is because the pruning mechanism used in the tree search of LAMP is more effective for sparser datasets. The figure also illustrates that, even by using 700 CPU cores, analyzing the whole genome dataset for 0.5 MAF limit is far beyond the ability of our current technique.

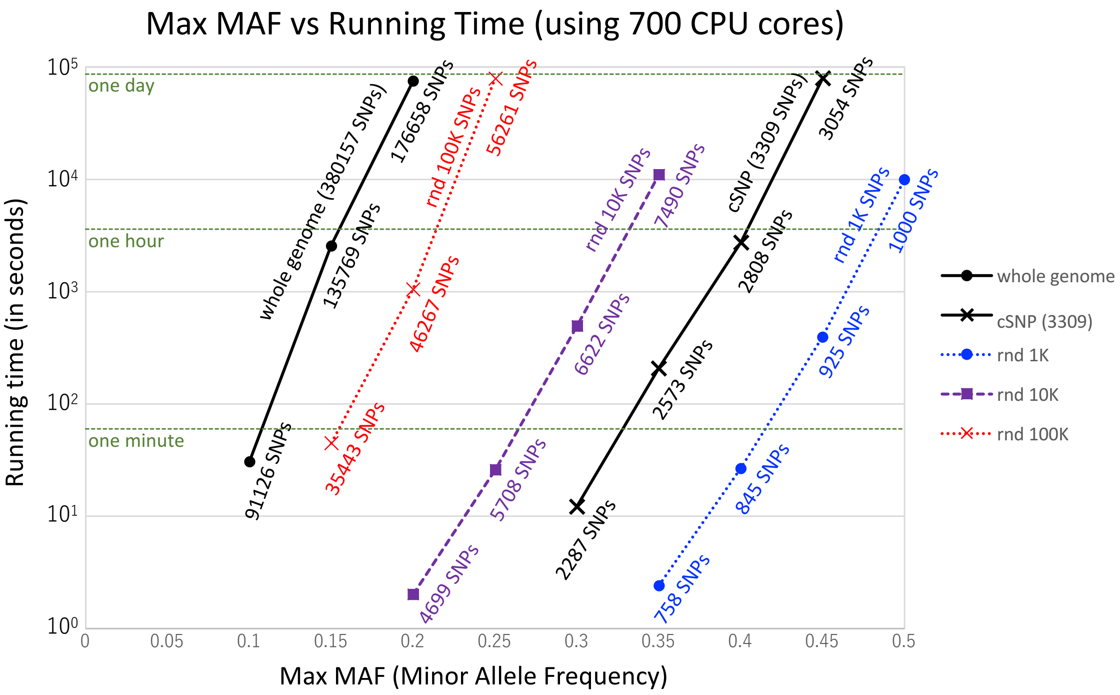

Figure S6: Max MAF vs Running time measured on a 700 CPU cores cluster. The leftmost line shows the runtime for the whole genome data. Other lines starting with "rnd" are using randomly sampled SNPs. The black (and cross) line shows the runtime only for the cSNPs.

References

[1] E. J. H. Chang. Echo Algorithms: Depth Parallel Operations on General Graphs. *IEEE Transactions on Software Engineering*, 8(4):391–401, 1982.

[2] E. H. Corder et al. Gene dose of apolipoprotein E type 4 allele and the risk of Alzheimer's disease in late onset families. *Science*, 261(5123):921–923, 1993.

[3] K. Dokladny, O. B. Myers, and P. L. Moseley. Heat shock response and autophagy: Cooperation and control. *Autophagy*, 11(2):200–213, 2015.

[4] Q. Li, Y. Liu, and M. Sun. Autophagy and Alzheimer's Disease. *Cellular and Molecular Neurobiology*, 37(3):377–388, 2017.

[5] F. Mattern. Asynchronous distributed termination—parallel and symmetric solutions with echo algorithms. *Algorithmica*, 5(1):325–340, 1990.

[6] S. Minato et al. A Fast Method of Statistical Assessment for Combinatorial Hypotheses Based on Frequent Itemset Enumeration. In *Proc of ECML/PKDD 2014*, 2014.

[7] V. A. Saraswat et al. Lifeline-based global load balancing. In *Proc. of PPoPP '11*, 2011.

[8] G. Sisti et al. Interaction between the inducible 70-kDa heat shock protein and autophagy: effects on fertility and pregnancy. *Cell stress & chaperones*, 20(5):753–758, 2015.

[9] R. E. Tarone. A modified Bonferroni method for discrete data. *Biometrics*, 46(2):515–522, 1990.

[10] A. Terada et al. Statistical significance of combinatorial regulations. *Proc Natl Acad Sci U S A.*, 110(32):12996–13001, 2013.

[11] A. Terada et al. LAMPLINK: detection of statistically significant SNP combinations from GWAS data. *Bioinformatics*, 32(22):3513–3515, 2016.

[12] The International HapMap Consortium. A haplotype map of the human genome. *Nature*, 437(7063):1299–1320, 2005.

[13] T. Uno, M. Kiyomi, and H. Arimura. LCM ver. 2: Efficient mining algorithms for frequent/closed/maximal itemsets. *Proc. IEEE ICDM'04 Workshop FIMI'04*, 126, 2004.

[14] J. A. Webster et al. Genetic control of human brain transcript expression in Alzheimer disease. *The American Journal of Human Genetics*, 84(4):445–458, 2009.

[15] D. M. Wolfe et al. Autophagy failure in Alzheimer's disease and the role of defective lysosomal acidification. *The European Journal of Neuroscience*, 37(12):1949–1961, 2013.
